# Multifunctional fiber-optic theranostic probe for closed-loop tumor photothermal therapy

**DOI:** 10.1038/s41377-026-02219-3

**Published:** 2026-04-27

**Authors:** Zesen Li, Zhuoran Li, Zhongyuan Cheng, Claudia Borri, Ambra Giannetti, Ni Lan, Junqiu Long, Wenwei Chen, Xiangran Cai, Jingge Yang, Bai-Ou Guan, Francesco Chiavaioli, Yang Ran

**Affiliations:** 1 Department of Gastrointestinal Surgery, the First Affiliated Hospital of Jinan University, Guangzhou, 510630 China; 2 Guangdong Provincial Key Laboratory of Optical Fiber Sensing and Communications, Institute of Photonics Technology, Jinan University, Guangzhou, 510632 China; 3 College of Physics & Optoelectronic Engineering, Jinan University, Guangzhou, 510632 China; 4 Medical Imaging Center, the First Affiliated Hospital of Jinan University, Guangzhou, 510630 China; 5 Institute of Applied Physics “Nello Carrara”, National Research Council of Italy (CNR), Sesto Fiorentino, 50019 Italy

**Keywords:** Biophotonics, Imaging and sensing

## Abstract

The combination of optical fiber and phototheranostic agents has emerged as a promising strategy to address the challenges of limited light penetration depth and systemic toxicity of nanomaterials. However, the multiplexing potential of fiber-optic probes remains underrated, resulting in enlarged incisions, repeated invasive procedures, and a lack of real-time therapeutic feedback. Herein, we propose a scheme for single‑fiber multifunctional integration leveraging wavelength division multiplexing technology. As a proof-of-concept, by co-immobilizing pH indicator, temperature indicator, and photothermal agent with non-overlapped excitation bands onto tapered optical fiber surface, a fiber-optic theranostic probe enabling closed-loop tumor photothermal therapy was developed. Pre-treatment, the probe can achieve tumor edge identification through revealing the tumor pH gradient. Intra-treatment, the photothermal agent can convert optical energy into heat for photothermal therapy, while simultaneous temperature monitoring enables precise thermal dose control. Post-treatment, rapid efficacy assessment can be achieved via real-time monitoring of the reversal of acidic tumor microenvironment. Animal experiments validate the excellent therapeutic efficacy and biocompatibility of the probe. This research opens new avenues for multifunctional fiber-optic theranostic platforms, where modular wavelength assignment enables customizable minimally invasive interventions and feedback monitoring, holding significant promise for both clinical practice and mechanistic exploration.

## Abstract (web summary)

Leveraging wavelength division multiplexing (WDM) strategy, the multifunctional fiber-optic theranostic probe enables pre-treatment tumor identification, intra-treatment photothermal therapy (PTT) with real-time dose monitoring, and post-treatment efficacy assessment, thereby establishing a complete closed-loop theranostic system.

## Graphical abstract

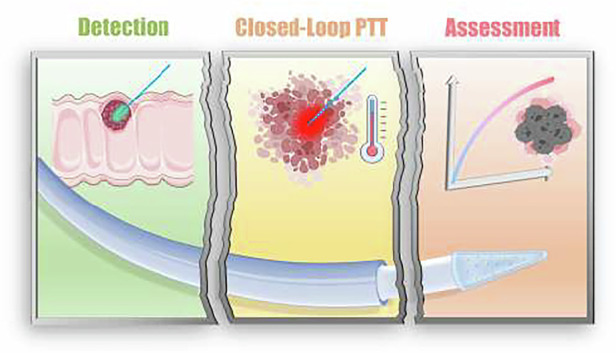

## Introduction

Cancer has become one of the most significant global public health challenges^1^. In 2022 only, approximately 20 million new cancer cases were diagnosed worldwide while nearly 10 million cancer-related deaths^2^. Motivated by this scenario, substantial efforts have been directed toward developing diagnostic and therapeutic methods with enhanced accuracy and efficacy. Theranostics, which integrates diagnostic and therapeutic functions into one spatially colocalized platform^3^, allows for immediate, targeted therapy after diagnosis and enables real-time monitoring of therapeutic dose and efficacy^4^, paving the way for personalized precision medicine. In the last decade, photo-theranostic has garnered widespread attention due to its advantages of excellent specificity, high spatiotemporal controllability, and non-ionizing nature^5,6^. However, several critical challenges prevent its clinical translation. One major obstacle is the inherently limited penetration depth of light (typically less than 10 mm) due to the scattering and absorption by tissues^6–8^. Although fluorescence dyes in the second near-infrared (NIR-II) window exhibited unprecedented penetration depth, their design and synthesis remain a great challenge^9^. Another significant limitation arises from the systemic toxicity caused by non-specific accumulation of nanomaterials on normal tissues and organs^10–12^.

Against this background, the combination of optical fiber and phototheranostic agents has emerged as a promising solution^6,8,11,13^. Flexible and compact optical fibers enable end-to-end light transmission with minimal loss, facilitating sensing and treatment of deep-seated tumors, including surgically inaccessible sites^14,15^. Furthermore, immobilizing phototheranostic agents on or within optical fibers effectively mitigates off-target toxicity through localized confinement. Benefitting from these features, optical fibers have been successfully applied for minimally invasive tumor therapy^16,17^ and in vivo biomarker monitoring^18,19^. Despite recent advances, the multiplexing potential of fiber-optic probes remains underrated. Current research remains limited to single-function-per-fiber implementations^20,21^ or suffers from inter-functional crosstalk^22,23^, which primarily arises from spectral overlap in the absorption or emission bands among the functional reagents used. Consequently, achieving multi-parameter monitoring or integrated theranostics demand multi-fiber configurations. This inevitably increases device rigidity and dimensions, decreasing compatibility for interventional techniques while elevating risks of tissue damage and post-treatment inflammation ^24^.

**Fig. 1:**
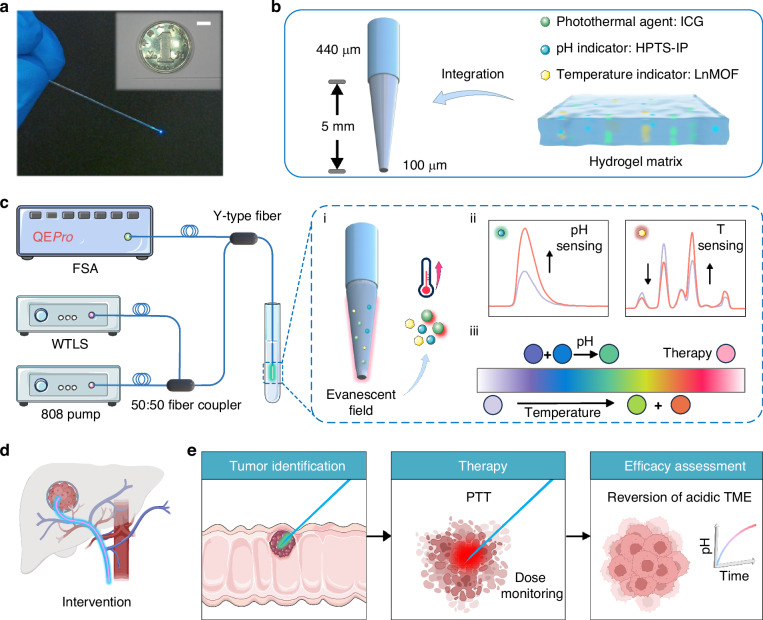
**Diagram of multifunctional fiber-optic theranostic probe**. **a** Photographs of the fiber-optic probe. The inset shows the size comparison between the probe and a 10-cent RMB coin. Scale bar = 5 mm. **b** pH indicator (HPTS-IP), temperature indicator (LnMOF), and photothermal agent (ICG) are co-encapsulated within a hydrogel matrix and immobilized onto tapered optical fiber surface. **c** Sketch of the experimental setup. i: ICG converts optical energy into heat; ii: Spectra for pH sensing and temperature sensing; iii: schematic diagram of the principle of wavelength multiplexing. The absorption and emission bands of the above agents do not overlap with each other. FSA: fluorescent spectral analyzer, WTLS: wavelength tunable light source. **d** Schematic diagram of the fiber-optic probe accessing tumor lesions via interventional procedures. **e** Schematic diagram of the fiber-optic probe used for closed-loop tumor management, including pre-treatment tumor identification, intra-treatment photothermal therapy (PTT) and dose monitoring, and post-treatment efficacy assessment

Inspired by the wavelength division multiplexing (WDM) technology that leverages wavelength separation to enhance the transmission capacity of a single optical fiber, which has been widely used in fiber-optic communication^25^, we propose in this work a scheme for fiber-optic multifunctional integration through modular wavelength assignment of photo-indicators&sensitizers to fully utilize the wavelength reservoir while suppress inter-functional crosstalk: (1) the UV–visible bands are employed for fluorescence probe excitation and emission to match the spectral characteristics of conventional fluorophores; (2) the NIR band, within the biological transparency window, is employed for photosensitizer excitation, ensuring compatibility with existing clinical therapeutic lasers and photosensitizers. Specifically, a pH indicator (HPTS-IP, derivative of 8-hydroxy-1,3,6-pyrene trisulfonic acid), a temperature indicator (LnMOF, lanthanide metal-organic framework material), and a photothermal agent (ICG, indocyanine) were co-encapsulated within a hydrogel matrix and immobilized onto tapered optical fiber surface (Fig. 1a, b). Crucially, the excitation bands of these agents do not overlap with each other. Consequently, the function of this probe can be switched on demand by using different excitation wavelengths (Fig. 1c). Clinically, this compact probe (diameter = 440 μm) can access tumor lesions via interventional procedures, enabling closed-loop tumor photothermal therapy with real-time feedback (Fig. 1d, e). Pre-treatment, the probe can achieve tumor edge identification through revealing the tumor pH gradient. Intra-treatment, the photothermal agent converts optical energy into heat for photothermal therapy (PTT), while simultaneous temperature monitoring enables precise thermal dose control. Post-treatment, rapid efficacy assessment can be achieved via real-time monitoring of the reversal of acidic tumor microenvironment (TME). This research establishes a paradigm shift for multifunctional fiber-optic theranostic platforms, offering significant potential for advancing both clinical practice and tumor mechanism research.

## Results and discussion

### Design and fabrication

A multifunctional fiber-optic probe was designed for tumor photothermal therapy and real-time monitoring of TME. The probe consists of a tapered optical fiber and a functional interface. The tapered optical fiber was chosen for its high mechanical stability and strong evanescent field^26^. It is fabricated following the established protocol^18^. Briefly, the distal end of a multimode optical fiber was etched into a tapered shape using hydrofluoric acid (HF) solution (Fig. S1a). The structure of the tapered optical fiber is shown in Fig. 1b and Fig. S1b. The taper length was set to 5 mm, because excessively short tapers risk light leakage due to unsatisfied total internal reflection conditions, which may excite tissue autofluorescence and degrade the signal-to-noise ratio (SNR). Conversely, overlong tapers are unmatched for localized sensing and therapy. The tip diameter was set to 100 μm based on our previous work^16^. While a smaller diameter strengthens the evanescent field and enhances fluorescence excitation/collection efficiency, it concurrently reduces the surface area available for indicator immobilization. Our previous comparative study using tapered fibers with different tip diameters demonstrated that the 100 μm diameter yielded the highest fluorescence intensity, confirming it as the optimal choice.

To implement the WDM-based fiber-optic theranostic probe, three agents were carefully selected, including HPTS-IP (pH indicator), LnMOF (temperature indicator), and ICG (photothermal agent). Critically, these agents exhibit non-overlapping absorption bands, which minimize crosstalk and enable accurate TME parameter sensing. Specifically, the absorption bands of HPTS-IP are located at 405 nm and 450 nm; the absorption band of LnMOF is located at 295 nm; the absorption band of ICG is located at 790 nm. This wavelength separation allows the fiber-optic probe to perform on-demand functions when excited by specific wavelengths. Furthermore, the selected pH indicator and temperature indicator are both ratiometric fluorescent probes. This feature mitigates interference from photobleaching, indicator concentration variations, and fiber morphology changes on the sensing results, facilitating the acquisition of accurate and reliable signals ^16,27^.

To achieve stable and biocompatible in vivo applications, the above agents were encapsulated within a sol-gel matrix composed of tetraethyl orthosilicate (TEOS) and glycidyl 3-(trimethoxysilyl)propyl ether (GLYMO). TEOS is one of the most widely used sol-gel precursors. However, sol-gel films prepared using pure TEOS suffer from severe cracking, leading to low film uniformity and high leaching rates. These defects compromise sensor stability and raise biocompatibility concerns. GLYMO, featuring a long carbon chain in its chemical structure, was employed as a co-precursor with TEOS to effectively mitigate cracking^28^. Furthermore, the surfactant Triton X-100 was introduced to reduce the film’s surface tension, further suppressing cracking and minimizing indicator leaching^29^. The functional sol-gel was applied to the tapered optical fiber surface via dip-coating method. While increasing dip-coating cycles could increase the film thickness and the number of immobilized functional reagents, thereby potentially improving sensing SNR and photothermal conversion performance, it would also prolong the diffusion time of target molecules (such as protons) within the hydrogel film, resulting in longer response times. Therefore, given that a single coating layer can provide sufficient performance in both sensing SNR and photothermal conversion, we consider it as the optimal choice for this application. SEM-EDS analysis confirmed the presence of Eu, Tb, and S elements (Eu and Tb originate from the LnMOF, while S derives from HPTS-IP and ICG) on the functionalized tapered optical fiber surface (Fig. S2a), indicating the successful modification. Atomic force microscopy (AFM) imaging revealed an average surface roughness of 26.7 nm, demonstrating a highly uniform functional gel coating with no observable film cracking (Fig. S2b).

### Performances of the fiber-optic theranostic probe

#### pH sensing

Extracellular acidification is a hallmark feature of the TME, playing a critical role in tumor initiation, progression, and metastasis^30^. The extracellular pH (pHe) inversely correlates with tumor severity and invasiveness^31^. Moreover, the acidic TME compromises the efficacy of diverse therapeutic approaches by inducing cancer stem cell phenotype, promoting immunosuppression, and impeding drug transmembrane delivery^32–34^. Therefore, precise pHe detection is clinically significant, facilitating both intra-treatment tumor identification and post-treatment efficacy assessment. It also serves as an indispensable tool in oncology research, providing fundamental mechanistic insights into tumor biology.

**Fig. 2:**
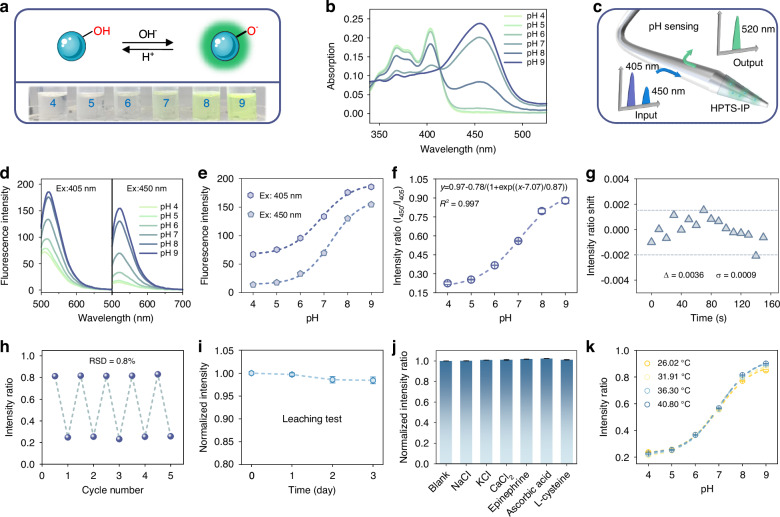
**pH sensing performance of the fiber-optic theranostic probe**. **a** pH sensing principle of HPTS-IP. The picture below shows the color change of HPTS-IP solution (10 μM in PBS buffer) with different pH values. **b** Absorption spectra of HPTS-IP solution (10 μM in PBS buffer) at varied pH values. **c** HPTS-IP is alternately excited by 405 nm and 450 nm light and emits fluorescence band peaks at 520 nm. **d**, **e** Fluorescence spectra and intensity calibration curves of the fiber-optic probe under 405 nm/450 nm excitation. **f** Ratiometric calibration curve in a pH range from 4.0 to 9.0. **g** Stability test in PBS buffer (pH 7.4) for 150 s. **h** Reversibility during cyclic immersion into pH 4.0/9.0 PBS buffers. **i** Leaching characteristics of HPTS-IP during 72-h immersion. Excitation light was blocked between the sampling points to minimize the interference of photobleaching. **j** Specificity of pH sensing, the concentration of the interfering substances was 100 μg/mL. **k** Temperature crosstalk assessment: pH calibration curves at different temperatures

In this study, we selected HPTS-IP as the pH indicator (Fig. 2a). It was synthesized through ion-pairing of HPTS with hexadecyl trimethyl ammonium bromide (Fig. S3). Compared to HPTS, HPTS-IP demonstrates higher hydrophobicity, large molecular volume, and reduced net charge. These properties minimize potential indicator leaching from the sol-gel film while reducing ionic strength interference on detection results^16^. As shown in Fig. 2b, HPTS-IP possesses two absorption peaks at 405 nm and 450 nm. The differential pH response of these peaks establishes HPTS-IP as well-suited for dual-excitation ratiometric fluorescence detection (Fig. 2c).

The pH sensing performance of the fiber-optic theranostic probe was evaluated in PBS buffers with a pH range from 4.0 to 9.0 at room temperature. As shown in Fig. 2d and e, emission intensity at 520 nm exhibited distinct trends when excited at 405 nm versus 450 nm. Notably, the 405 nm-excited fluorescence response to pH deviated from absorption spectral behavior. This phenomenon stems from differences between the absorption spectra of fixed-state versus free-state indicators, coupled with pH-dependent variations in fluorescence quantum yield^16,35^. The relationship between pH value and fluorescence intensity ratio (I_450_/I_405_) was accurately fitted using the Boltzmann model (Fig. 2f). For stability assessment, the probe was immersed in PBS buffer (pH 7.4) with continuous spectral recording for 150 s. The standard deviation (σ) of the intensity ratio was calculated to be 0.0009 (Fig. 2g). Applying the resolution equation R = 3σ/s (R: pH resolution; s: sensitivity as the calibration curve slope)^36^, the probe achieved a resolution of 0.013 pH units within the linear pH range from 6.0 to 8.0, which cover physiological pH values in both normal and tumor tissues.

The response time of pH sensing is shown in Fig. S4a. As the pH increased from 6.0 to 8.0, the response time τ_1_ = 3.2 s, while the response time τ_2_ = 12.8 s was observed as the pH decreased from 8.0 to 6.0. To assess the reversibility, the fiber-optic probe was alternately immersed in PBS buffers at pH 4.0 and 9.0. Over five cycles, the intensity ratio demonstrated high consistency with a relative standard deviation (RSD) of 0.8% (Fig. 2h). Additionally, the probe was immersed in PBS buffer for 72 h to evaluate leaching characteristics. A decrease of 1.6% in fluorescence intensity was observed, indicating minimal leaching of HPTS-IP from the functional film (Fig. 2i). To further evaluate the sensing reliability in complex physiological environments, the response was tested in PBS buffers containing potential interfering substances. As shown in Fig. 2j, no significant change in the intensity ratio, confirming excellent selectivity. Moreover, temperature crosstalk was also assessed by changing temperature from 26 to 41 °C. The sensor exhibited negligible temperature crosstalk within the pH range from 4.0 to 7.0 (Fig. 2k). For measurements at pH > 7.0, the capability of temperature monitoring of the developed fiber probe can provide built-in compensation for pH sensing, thereby ensuring the pH sensing accuracy.

#### Temperature sensing

Photothermal therapy utilizes light energy to elevate tissue temperature, achieving localized photocoagulation. When tissues are heated to the sub-coagulation range (43–55 °C) or the coagulation range (55–100 °C), cells rapidly die due to protein denaturation and cell membrane damage^37,38^. Furthermore, PTT can reshape the tumor-suppressive microenvironment through different mechanisms including enhanced blood perfusion, reduced tumor interstitial pressure, and induced immunogenic cell death (ICD)^39–41^. However, excessive heating (either in temperature or duration) may not only damage adjacent normal tissues but also induce vascular collapse and impair blood perfusion^39^. Therefore, combining with real-time monitoring of temperature and key parameters of TME remodeling (pH) can prevent undertreatment or excessive damage. This approach also provides critical information for determining the optimal photothermal dose (temperature × time), thereby guiding the optimization of treatment strategies.

**Fig. 3:**
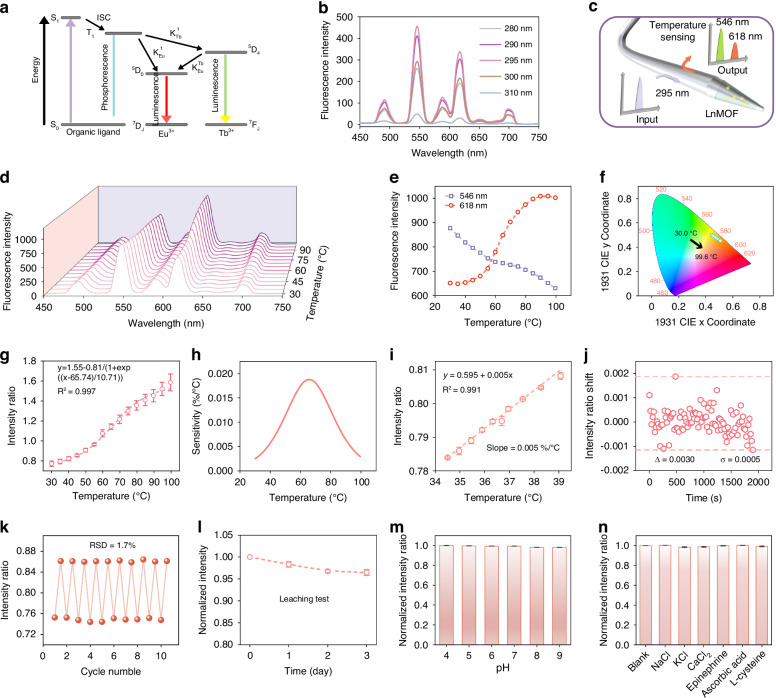
**Temperature sensing performance of the fiber-optic theranostic probe**. **a** The luminescence mechanism of LnMOF. **b** Fluorescence spectra of the probe under excitation of different wavelengths. **c** LnMOF is excited by 295 nm light and emits two fluorescence bands with peaks at 546 nm and 618 nm, respectively. **d**, **e** Fluorescence spectra and intensity curve of the probe at different temperatures under 295 nm light excitation. **f** 1931 CIE chromaticity diagram, showing the change of fluorescence color with increasing temperature. **g** Calibration curve in the temperature range of 30 ~ 100 °C. **h** The slope of the calibration curve. **i** Temperature sensing calibration curve in the range near body temperature. **j** Stability test in PBS buffer (pH 7.4) for 0.5 h. **k** Reversibility during cyclic immersion in PBS buffer at 26 °C and 46 °C. **l** Leaching characteristics of LnMOF during 72-h immersion. Excitation light was blocked between the sampling points. **m** pH independence of temperature sensing. **n** Specificity of temperature sensing, the concentration of the interfering substances was 100 μg/mL

Here, we selected LnMOF as the temperature indicator. Due to the parity-forbidden nature of *f*–*f* transitions, lanthanide ions typically exhibit weak light absorption capacity, while coordination with organic ligands can break the inversion symmetry around the ions, thereby enhancing transition probabilities and efficient emission^42^. The luminescence mechanism of LnMOF is illustrated in Fig. 3a. The contrast thermal dependence of the emission intensities of Tb^3+^ (^5^D_4_ → ^7^F_5_) and Eu^3+^ (^5^D_0_ → ^7^F_2_) is primarily due to the thermally enhanced energy transfer from Tb^3+^ to Eu^3+^, thereby rendering the LnMOF suitable for ratiometric temperature sensing^43^. Since the optimal excitation wavelength for LnMOF cannot be directly determined via UV–visible absorption spectroscopy, this study employed excitation light of different wavelengths to test the fiber-optic probe. The results (Fig. 3b) show that the fluorescence intensity reaches its maximum with an excitation wavelength of 295 nm. Therefore, this wavelength was used for LnMOF excitation in subsequent experiments (Fig. 3c).

The temperature sensing performance of the fiber-optic theranostic probe was evaluated over a wide range of 30–100 °C. Results show that as temperature increases, the fluorescence intensity at 618 nm (I_618_) exhibits an S-shaped increase, while the intensity at 546 nm (I_546_) decreases (Fig. 3d and e). Visualization using the 1931 CIE chromaticity diagram reveal a gradual shift in fluorescence color from yellow to orange-red with increasing temperature (Fig. 3f). The relationship between the fluorescence intensity ratio (I_618_/I_546_) and temperature can be described using the Boltzmann model (Fig. 3g). Differentiation of this fitting function shows that the temperature sensitivity exhibits a peak at 66 °C (Fig. 3h), indicating the probe’s capability for high-precision temperature monitoring during photothermal therapy. Furthermore, the probe’s temperature response was characterized within the physiologically relevant range of 34.5–39 °C. A linear relationship was observed between temperature and the fluorescence ratio (I_618_/I_546_), yielding a slope of 0.005 (Fig. 3i). Consequently, the temperature resolution is calculated to be 0.3 °C, satisfying the requirements for in vivo temperature monitoring.

The response time of temperature sensing is shown in Fig. S4b. As the temperature increased from 26 to 60 °C, the response time τ_1_ = 2.0 s, while the response time τ_2_ = 0.6 s was observed as the temperature decreased from 60 to 26 °C. To verify the reversibility of temperature sensing, the fiber-optic probe was alternately immersed in PBS buffers at 26 °C and 46 °C. Over 10 cycles, the fluorescence intensity ratio (I_618_/I_546_) exhibited excellent consistency (Fig. 3k) with a relative standard deviation of 1.7%, demonstrating outstanding reversibility. In addition, to evaluate the leaching stability of LnMOF, the probe was immersed in PBS buffer (pH 7.4) for 72 h. Results show a decrease of 3.5% in fluorescence intensity after 72 h (Fig. 3l), indicating minimal leaching of LnMOF from the functional membrane. To assess the pH independence of temperature sensing, the probe was tested in buffers across a pH range from 4.0 to 9.0. As shown in Fig. 3m, pH variations had a negligible impact on the fluorescence intensity ratio. To further evaluate reliability in complex physiological environments, the probe’s response was tested in PBS buffers containing potential interfering substances. As shown in Fig. 3n, the temperature sensing results showed no significant interference, confirming excellent specificity.

Tissue autofluorescence poses a known limitation to fluorescence imaging quality and specificity in living tissues^44^. To evaluate its potential impact on the sensing accuracy of the developed fiber-optic probe, a tapered optical fiber without the functional coating was implanted into both tumor and normal tissues. The fluorescence spectra under 295 nm, 375 nm, 450 nm, and 808 nm excitation were recorded. As shown in Fig. S5, no fluorescence emission was observed. This negligible autofluorescence interference can be attributed to the shallow penetration depth of the evanescent field, where the concentration of the immobilized pH and temperature indicators is significantly higher than that of potential interfering molecules in the tissue, thereby guaranteeing a high SNR even with minimal excitation power.

At last, the potential impact of prolonged 808 nm laser irradiation on the stability and accuracy of pH and temperature sensing were evaluated by measuring the fluorescence spectra after continuous irradiation for 10, 30, and 60 min. The results indicated gradual decrease in the fluorescence intensities of both pH and temperature sensing (Fig. S6). This could stem from the accelerated leaching of functional indicators under elevated temperature. Notably, the intensity ratio of both pH and temperature sensing remained stable throughout the irradiation period, confirming the crucial value of the ratiometric detection strategy in ensuring long-term monitoring reliability.

#### Photothermal conversion

**Fig. 4:**
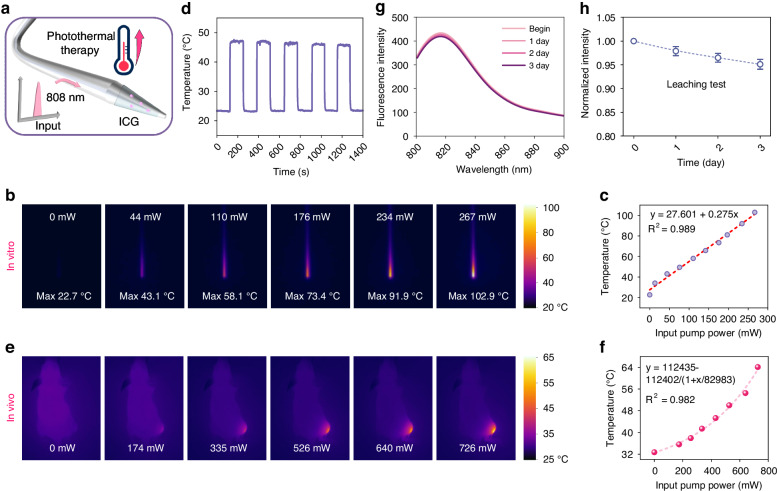
**Photothermal conversion performance of the fiber-optic theranostic probe**. **a** ICG is excited by an 808 nm pump laser and generates heat. **b**, **c** Thermal images and maximum temperatures of the probe at different pump powers. **d** Photothermal cycling stability over 5 heating/cooling cycles. **e**, **f** The thermal images of mice and the maximum temperature of the tumor region with increasing pump power. **g**, **h** Leaching characteristics of ICG during 72-h immersion. Excitation light (785 nm) was blocked between the sampling points

In this study, ICG was chosen as the photothermal agent due to its narrower absorption bandwidth compared to conventional inorganic photothermal agents. As shown in Fig. S7, ICG exhibits an absorption peak at 790 nm with negligible absorption below 650 nm. This characteristic minimizes interference with temperature and pH sensing spectra, facilitating wavelength multiplexing. Experimentally, ICG was excited using an 808 nm pump laser (Fig. 4a). Under only 267 mW excitation power, the probe heated itself to 102.9 °C (Fig. 4b and c), confirming efficient photothermal conversion capability. Therefore, the required pump power for fiber-optic PTT is significantly lower than that typically employed in nanomaterial-based systems (>1 W), making it more cost-effective while reducing potential side effects. Photothermal stability was further evaluated through 5 heating/cooling cycles. As shown in Fig. 4d, the temperature elevation amplitude during pumping remained stable, indicating excellent photothermal stability. To validate applicability under physiological conditions, in vivo experiments were conducted using tumor-bearing mice. As shown in Fig. 4e and f, the temperature of tumor region increased progressively with rising pumping power. It is worth noting that the significant temperature difference observed between the probe self-heating in air and heating within the tumor region can be attributed to the reason that thermal imaging can only detect surface temperatures, and the temperature gradient established during internal tissue heating inevitably results in tissue surface temperatures being lower than the actual temperature of the fiber probe surface. At Last, to evaluate the leaching stability of ICG, the probe was immersed in PBS buffer (pH 7.4) for 72 h. Results show a decrease of 5.9% in fluorescence intensity after 72 h (Fig. 4g, h), indicating minimal leaching of ICG.

### In vivo experiments

#### Tumor identification

**Fig. 5:**
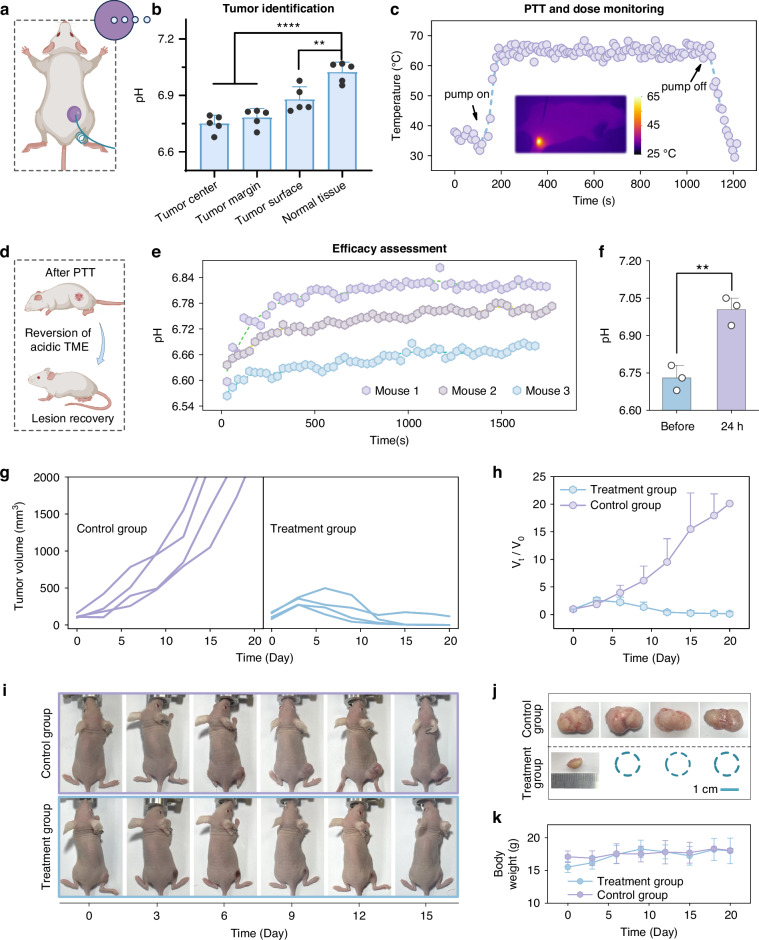
**In vivo validation**. **a**, **b** Comparison of pH values measured at the tumor center, tumor margin, tumor surface, and normal tissue (*n* = 5). **p < 0.01, ****p < 0.0001, unpaired t-test. **c** Real-time temperature monitoring during PTT. The inset is the thermal image of a mouse during PTT. **d**, **e** Real-time monitoring of pH in TME within 30 minutes post-treatment. **f** Comparison of the pH values of TME before and 1 day after treatment. **g**, **h** Changes in tumor volume in the control group and treatment group within 20 days after treatment. **i** Photos of mice in the control group and the treatment group within 15 days after treatment. **j** Comparison of tumor volumes between the two groups on the 20th day after treatment. **k** Body weight changes of mice in the two groups within 20 days after treatment

To validate the reliability of the fiber-optic theranostic probe in physiological environments, we established subcutaneous colorectal cancer xenograft models in mice. Prior to PTT, the probe’s pH-sensing capability was employed to distinguish between tumor and normal tissues. As shown in Fig. 5a, the probe was inserted into both tumor tissue and adjacent normal tissue, with fluorescence spectra recorded after 1 min. Results revealed a statistically significant difference (*p* < 0.0001) in measured pH values between healthy and tumor tissues (Fig. 5b), demonstrating the probe’s ability to differentiate tumor from normal tissue. Furthermore, a still significant difference in measured pH values (*p* < 0.01) was also observed between the tumor surface and normal tissue, confirming the probe’s capability for precise tumor edge identification. This functionality is crucial for ensuring both therapeutic efficacy and safety of PTT, as it enables accurate treatment boundary definition to prevent collateral damage to surrounding healthy tissues.

#### Photothermal therapy and dose monitoring

After determining the tumor’s precise location and size, the fiber-optic probe was inserted into the tumor center for photothermal therapy. Since temperature monitoring is confined to the tapered optical fiber surface, the fiber should be heated to a higher temperature to ensure the entire tumor reaches the sub-coagulation temperature. As shown in Fig. 5c, throughout the 15-min treatment, the fiber temperature was actively maintained at 65 °C to achieve comprehensive tumor tissue ablation while mitigating potential side effects. The decision to set the fiber temperature at 65 °C was based on the observation that when the fiber was maintained at this temperature in tumors of approximately 100 mm^3^, thermal imaging confirmed that the tumor edge reached an effective therapeutic temperature of approximately 45 °C. Looking forward, given the inherent electromagnetic immunity of optical fibers, the developed system could be readily integrated with MRI thermometry in an MR environment to achieve more accurate temperature monitoring of the tumor edge.

#### Efficacy assessment

Following photothermal therapy, the pH sensing functionality of the fiber-optic theranostic probe was further deployed for efficacy assessment. As shown in Fig. 5d and e, the measured pH values of TME increased by 0.12, 0.14, and 0.22 units within 30 min post-treatment in 3 mice, respectively. This phenomenon can be primarily attributed to both photothermally enhanced blood perfusion, which facilitates hydrogen ion clearance, as well as a reduction in acid production resulting from cancer cell death^39^. Reassessment at 24 h post-treatment demonstrated significant pH elevation compared to pre-treatment baseline (*p* < 0.01; Fig. 5f). Given the established correlation between acidic TME and malignant progression, this pH reversal serves as a biomarker for rapid therapeutic evaluation.

To further evaluate the efficacy of fiber-optic PTT, the tumor volume and body weight of mice were monitored every 3 days. As shown in Fig. 5g–i, PTT-treated mice developed eschar at tumor sites that spontaneously detached after ~15 days, with subsequent replacement by neo-healing tissue. In contrast, tumors of the control group exhibited progressive growth (euthanasia was performed when tumor volume exceeded 2000 mm^3^, followed by tumor tissue collection). On day 20, all surviving mice were euthanized for comparison of tumor volume. Results (Fig. 5j) demonstrated complete tumor regression in 3 of 4 treated mice, with the remaining mouse showing significant tumor growth suppression, robustly validating the antitumor efficacy of fiber-optic PTT. Moreover, both groups maintained stable body weights throughout the 20-day post-treatment period (Fig. 5k), confirming the biosafety of this therapeutic approach.

**Fig. 6:**
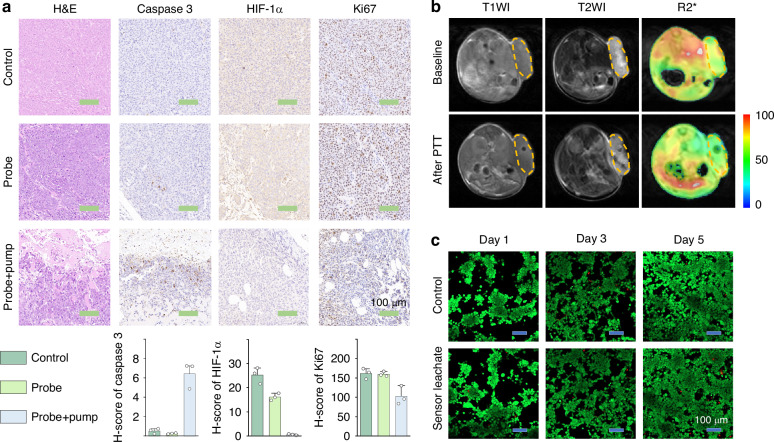
**Efficacy and biocompatibility analyses**. **a** H&E staining and immunohistochemistry (Caspase 3, HIF-1α, and Ki67) of the control group, implantation group (fiber implantation without laser excitation), and treatment group. **b** MRI image: comparison of tumors before (baseline) and 2 h after treatment. **c** Live/dead staining of HCT116 cells co-cultured with culture medium soaked by the probe for 24 h (sensor leachate group) and pure culture medium (control group). Live cells were stained green by Calcein-AM, while dead cells were stained red by PI

To further investigate the therapeutic efficacy of fiber-optic PTT, histological and immunohistochemical analyses were performed on tumor tissues harvested 24 hpost-treatment (Fig. 6a). H&E staining revealed no significant apoptotic/necrotic areas or inflammatory cell infiltration in either control or sham-treated groups (fiber implantation without laser excitation), whereas the treatment group exhibited multifocal necrosis with hemorrhagic regions. Immunohistochemical analysis demonstrated upregulated expression of Caspase-3 (apoptosis biomarker) and downregulated expression of Ki67 (proliferation biomarker) in treated tumors, confirming that fiber-optic PTT simultaneously induces tumor cell apoptosis and suppresses proliferation. Notably, treated tumors showed reduced expression of hypoxia-inducible factor HIF-1α. As a crucial regulatory factor, HIF-1α undergoes rapid proteasomal degradation under normoxia but is upregulated in hypoxic microenvironments. It transcriptionally regulates more than 100 downstream genes directly or indirectly, critically influencing tumor metabolism, metastasis, angiogenesis, and therapy resistance^45^. The observed HIF-1α downregulation indicates ameliorated tumor hypoxia, which was also verified by magnetic resonance imaging (Fig. 6b and Fig. S8). Collectively, these findings demonstrate that the fiber-optic theranostic probe exerts multimodal and excellent anti-tumor effects.

At last, to evaluate biocompatibility, the fiber-optic theranostic probe was immersed in culture medium for 24 h to prepare leachate, which was subsequently used for cell culture to evaluate viability (CCK-8 assay) and proliferation status (Calcein AM/PI live-dead dual staining). CCK-8 results (Fig. S9) showed no significant differences in cell viability between experimental and control groups on days 1, 3, and 5, confirming excellent cytocompatibility. Live-dead staining further validated these findings (Fig. 6c): at all time points, the majority of cells exhibited green fluorescence (live cells) with minimal red fluorescence (dead cells). In addition, Organ biocompatibility (heart, liver, spleen, lung, and kidney) was also assessed. In the experiment, the probes were inserted into the tumor tissues of mice for 1 h, and the probes in the treatment group were excited by an 808 nm pump for 15 min, while those probes in the implantation group were not. After 24 h, organs were harvested for H&E staining analysis. Results (Fig. S10) revealed no significant apoptotic/necrotic areas or inflammatory cell infiltration in either implantation or treatment groups compared to controls, demonstrating high histocompatibility of the theranostic probe.

## Conclusion

In this study, we developed a multifunctional fiber-optic theranostic probe for closed-loop tumor photothermal therapy by co-immobilizing absorption spectrally separate agents, including a pH indicator (HPTS-IP), a temperature indicator (LnMOF), and a photothermal agent (ICG) onto tapered optical fiber surface. Compared to nanomaterial-dependent strategies, this approach effectively addresses limitations of light penetration depth and systemic toxicity. Simultaneously, compared to reported fiber-optic probes, our design achieves unprecedented functional integration, thereby avoiding surgical incision enlargement, eliminating repeated invasive procedures, and resolving the absence of real-time therapeutic feedback. In vitro experiments validated the exceptional pH/temperature sensing ability and photothermal conversion efficiency of the probe. Subsequent in vivo studies demonstrated various probe functions at different stages of treatment, including: tumor edge identification, photothermal therapy, thermal dose monitoring, and efficacy assessment. Results conclusively demonstrated the effectiveness of fiber-optic PTT.

Looking forward, the presented integration strategy can be readily translated to flexible polymer and hydrogel optical fibers^46–49^. Their exceptional mechanical compliance and biocompatibility^50^ make them ideal for minimizing tissue damage and enabling the future development of long-term implantable devices capable of continuous monitoring and therapy. In addition, given that real-time monitoring of multiple parameters is crucial for investigating pathophysiological processes regulated by complex biological interactions^51–53^, the functional capacity of a single optical fiber can be further enhanced by fully leveraging the spectral resources and employing multipeak fitting algorithms^54^ to address potential emission bands overlap as the number of integrated functions increases. We anticipate that this high-performance yet facile fabrication strategy for multifunctional integration of fiber-optic probes will hold substantial promise for both clinical practice and mechanistic research in oncology and beyond, paving the way for new clinical equipment.

## Materials and methods

### Materials

Ethanol (EtOH), sulfuric acid (H_2_SO_4_, 98%), hydrochloric acid (HCl), hydrofluoric acid (HF), sodium hydroxide (NaOH), N,N-dimethylformamide (DMF), and hydrogen peroxide (H_2_O_2_, 30%) were purchased from Guangzhou Chemical Reagent Factory (Guangzhou, China). Indocyanine green (ICG) and glycidyl 3-(trimethoxysilyl)propyl ether (GLYMO) were purchased from Macklin Biochemical Technology Co., Ltd. (Shanghai, China). Phosphate buffered saline (PBS) tablets were purchased from Beijing Solarbio Science & Technology Co., Ltd. (Beijing, China). Hexadecyl trimethyl ammonium bromide (CTAB) and 8-hydroxy-1,3,6-pyrene trisulfonic acid (HPTS) were purchased from Shanghai Bide Pharmaceutical Technology Co., Ltd. (Shanghai, China). Terbium(III) nitrate hexahydrate (Tb(NO_3_)_3_·6H_2_O), europium(III) nitrate hexahydrate (Eu(NO_3_)_3_·6H_2_O), 1,3,5-benzenetricarboxylic acid (BTC), sodium acetate, tetraethyl orthosilicate (TEOS), Triton X-100, Calcein-AM, and propidium iodide (PI) were purchased from Shanghai Aladdin Biochemical Technology Co., Ltd. (Shanghai, China). Dulbecco’s Modified Eagle Medium (DMEM) was purchased from Procell Life Science & Technology Co., Ltd. (Wuhan, China). Fetal bovine serum (FBS) was purchased from Shanghai ExCell Bio, Inc. (Shanghai, China). The CCK-8 assay kit was purchased from Dojindo Molecular Technologies, Inc. (Shanghai, China). All reagents were of analytical grade and were used without further purification. Multimode optical fiber (core diameter: 400 μm, cladding diameter: 440 μm, numerical aperture: 0.22) was purchased from Thorlabs, Inc.

### Synthesis of LnMOF

LnMOF was synthesized according to the published procedures^43^. First, using DMF/H_2_O (5:1, v/v) as the solvent, the following solutions were prepared: 274 mM Tb(NO_3_)_3_·6H_2_O, 2.6 mM Eu(NO_3_)_3_·6H_2_O, 1.3 mM BTC, and 1 mM sodium acetate. Then, 0.3 mL of each of the above-prepared solutions was added to a glass flask, followed by adding 2.4 mL DMF/H_2_O (5:1, v/v) solution. Next, the flask was placed in an oil bath at 60 °C and heated for 24 h with continuous stirring, and a large amount of precipitation can be observed. After the reaction completes, LnMOF was collected by centrifugation and washed 5 times with water. Finally, the LnMOF was vacuum-dried at 40 °C overnight.

### Preparation of functional sol-gel

First, 896.6 mg TEOS, 102.2 mg GLYMO, 1.2 mL ethanol, 0.4 mL of 0.1 M HCl solution, and 0.2 mL of 2 mM Triton X-100 aqueous solution were added into a glass flask. The mixture was aging at room temperature for 2 days. Subsequently, 1 mg HPTS-IP, 1 mg ICG, and 10 mg LnMOF were added to the above solution (Optimization experiments for the concentrations of the three functional indicators are shown in Fig. S11). Then, the mixture was stirred for 30 min before being deposited onto the optical fiber surface.

### Fabrication of fiber-optic theranostic probe

The functional sol-gel was deposited onto the tapered optical surface via dip-coating method. First, the tapered optical fiber was immersed in freshly prepared piranha solution (H_2_SO_4_/H_2_O_2_, 3:1, v/v) for 1 h to clean and hydroxylate the fiber surface. Then, rinse the fiber in deionized water for 5 min to remove residual piranha solution. Next, the fiber was immersed in the aforementioned functional sol-gel for 30 min. Finally, the optical fiber was cured in a nitrogen-purged oven at 60 °C for 12 h. The obtained fiber-optic theranostic probe was stored at 4 °C in a dark environment before used.

### Experimental setup

The experimental setup is shown in Fig. 1c. The fiber-optic theranostic probe is connected to both the excitation light source and the fluorescent spectral analyzer (QE pro, Ocean optic) via a Y-type fiber. The output light from the wavelength-tunable light source (customized by Beijing Zhuoli Hanguang Instrument Co., Ltd.) is used for fluorescent probe excitation. Thus, by changing the output light wavelength, on-demand switching of the sensing functions can be achieved.

### Cell line and culture conditions

The human colon cancer cell line HCT116 was purchased from the American Type Culture Collection (ATCC). Cells were cultured in DMEM medium supplemented with 10% fetal bovine serum and 1% penicillin-streptomycin, and maintained at 37 °C with 5% CO_2_.

### Animals

All animal experiments were approved by the Laboratory Animal Welfare and Ethics Committee of Jinan University (Approval number: IACUC-20241111-02). Four- to six-week-old female BALB/c nude mice (BALB/cJGpt-Foxn1nu/Gpt) were purchased from GemPharmatech (Nanjing, China) and housed under specific pathogen-free (SPF) conditions.

### Establishment of xenograft models of colorectal cancer

HCT116 cells (1 × 10^5^ cells) were suspended in 20 μL of Matrigel and inoculated subcutaneously into the right flank of nude mice. Experiments were initiated when the tumor volumes reached approximately 100 mm^3^.

## Supplementary information

Supplementary material

## Data Availability

All the data supporting this study are available upon request to the corresponding authors.
