# Supplementary material for: Multifunctional fiber-optic theranostic probe for closed-loop tumor photothermal therapy
Source: Light Sci Appl. 2026 Apr 27;15:216. doi: 10.1038/s41377-026-02219-3 (PMC13121746; doi:10.1038/s41377-026-02219-3)
Supplement: Supplementary file 1 — Supplementary material [file 41377_2026_2219_MOESM1_ESM.docx]

**Supplementary Information**

**Multifunctional fiber-optic theranostic probe for closed-loop tumor photothermal therapy**

Zesen Li ^123#^, Zhuoran Li ^23#^, Zhongyuan Cheng ^4#^, Claudia Borri ^5^, Ambra Giannetti ^5^, Ni Lan ^23^, Junqiu Long ^23^, Wenwei Chen ^23^, Xiangran Cai ^4^, Jingge Yang ^1,^*, Bai-Ou Guan ^23,^*, Francesco Chiavaioli ^5,^*, and Yang Ran ^23,^*

^1^ Department of Gastrointestinal Surgery, the First Affiliated Hospital of Jinan University, Guangzhou, 510630, China.

^2^ Guangdong Provincial Key Laboratory of Optical Fiber Sensing and Communications, Institute of Photonics Technology, Jinan University, Guangzhou, 510632, China.

^3^ College of Physics & Optoelectronic Engineering, Jinan University, Guangzhou, 510632, China.

^4^ Medical Imaging Center, the First Afﬁliated Hospital of Jinan University, Guangzhou, 510630, China.

^5^ Institute of Applied Physics “Nello Carrara”, National Research Council of Italy (CNR), 50019 Sesto Fiorentino (FI), Italy

# These authors contributed equally to this work.

*Corresponding authors: J. Y. (dukeyjg@126.com); B.-O. G. ([tguanbo@jnu.edu.cn](mailto:tguanbo@jnu.edu.cn)); F. C. (f.chiavaioli@ifac.cnr.it); Y. R. ([tranyang@jnu.edu.cn](mailto:tranyang@jnu.edu.cn)).

**CCK-8 assay**

First, the fiber-optic theranostic probe was sterilized by exposure to ultraviolet (UV) light for 30 minutes. Subsequently, it was immersed in cell culture medium for 24 hours to prepare the leachate. The leachate was then filtered using a 0.22 μm microporous membrane. HCT116 cells were inoculated into a 96-well plate at a density of 10^4^ cells per well. After 24 hours, the culture medium in the 96-well plate was aspirated. The prepared leachate was added to the experimental group wells. Meanwhile, a control group was established using fresh, normal culture medium. At the selected time points (Days 1, 3, and 5), the culture medium was aspirated and replaced with CCK-8 solution. The cells were then incubated at 37 °C with 5% CO_2_ for 1 hour. Finally, the optical density (OD) at 450 nm for each well was measured. The OD value is proportional to cell viability.

**Live/dead staining**

HCT116 cells were cultured according to the method described above. At the same time points (Days 1, 3, and 5), the culture medium was aspirated, and the cells were washed with PBS buffer to remove residual medium. Afterwards, the cells were incubated with a Calcein-AM/PI solution for 30 minutes. Subsequently, the staining results were observed under a laser scanning confocal microscope. Viable cells were stained green by Calcein-AM, while dead cells were stained red by PI.

**Prepared and characterization of tapered optical fiber**

**
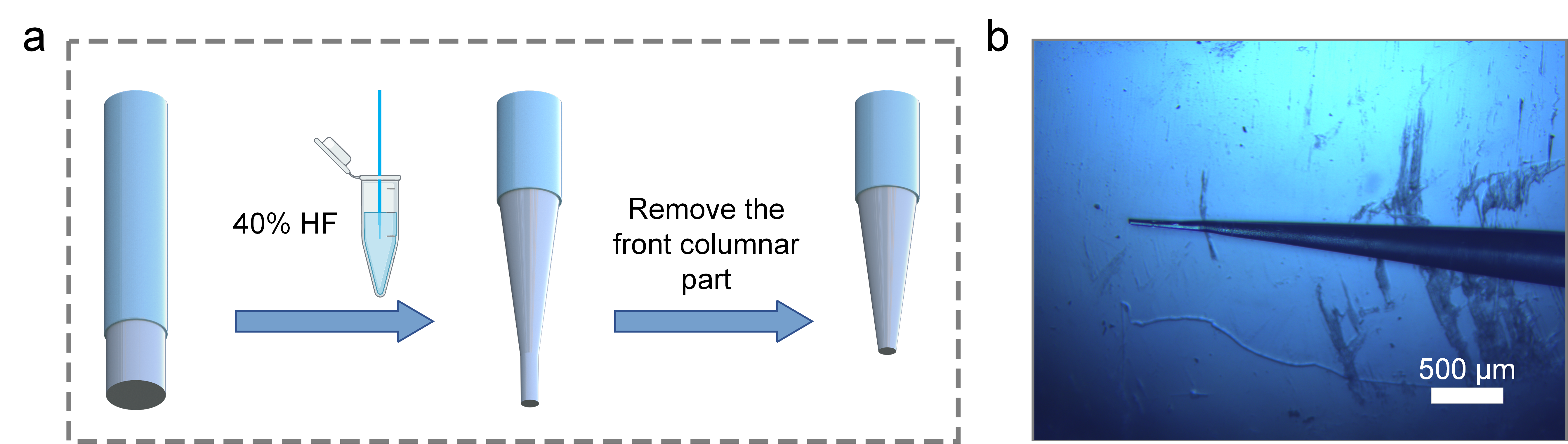
**

**Fig. S1.** Preparation and characterization of tapered optical fiber. (a) The preparation steps: (1) Remove the coating layer from the front section of 2 mm of the optical fiber. (2) Immerse the front 7 mm of the fiber in a 40% HF acid solution for 150 minutes. The quartz-based fiber core and cladding will be etched, while the acrylic-based coating layer will not. As etching progresses, the fiber gradually transforms into a tapered columnar structure. (3) After etching is complete, thoroughly rinse with deionized water to remove residual HF acid solution. (4) Finally, remove the front columnar portion of the optical fiber. (b) Microscope image of the tapered optical fiber.

**Characterization of the fiber-optic theranostic probe**

**
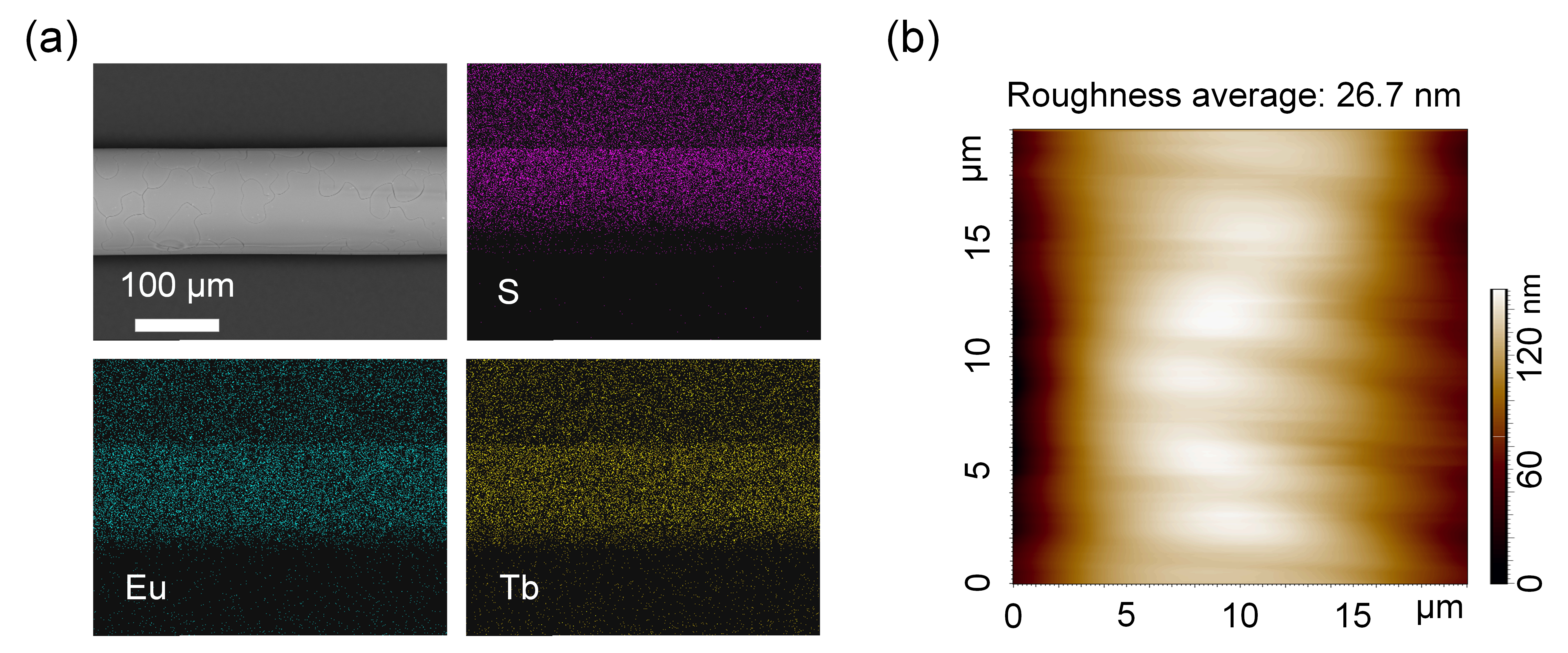
**

**Fig. S2.** Characterization of the fiber-optic theranostic probe. (a) SEM image and EDS mapping images. (b) AFM image.

**Synthesis of HPTS-IP**


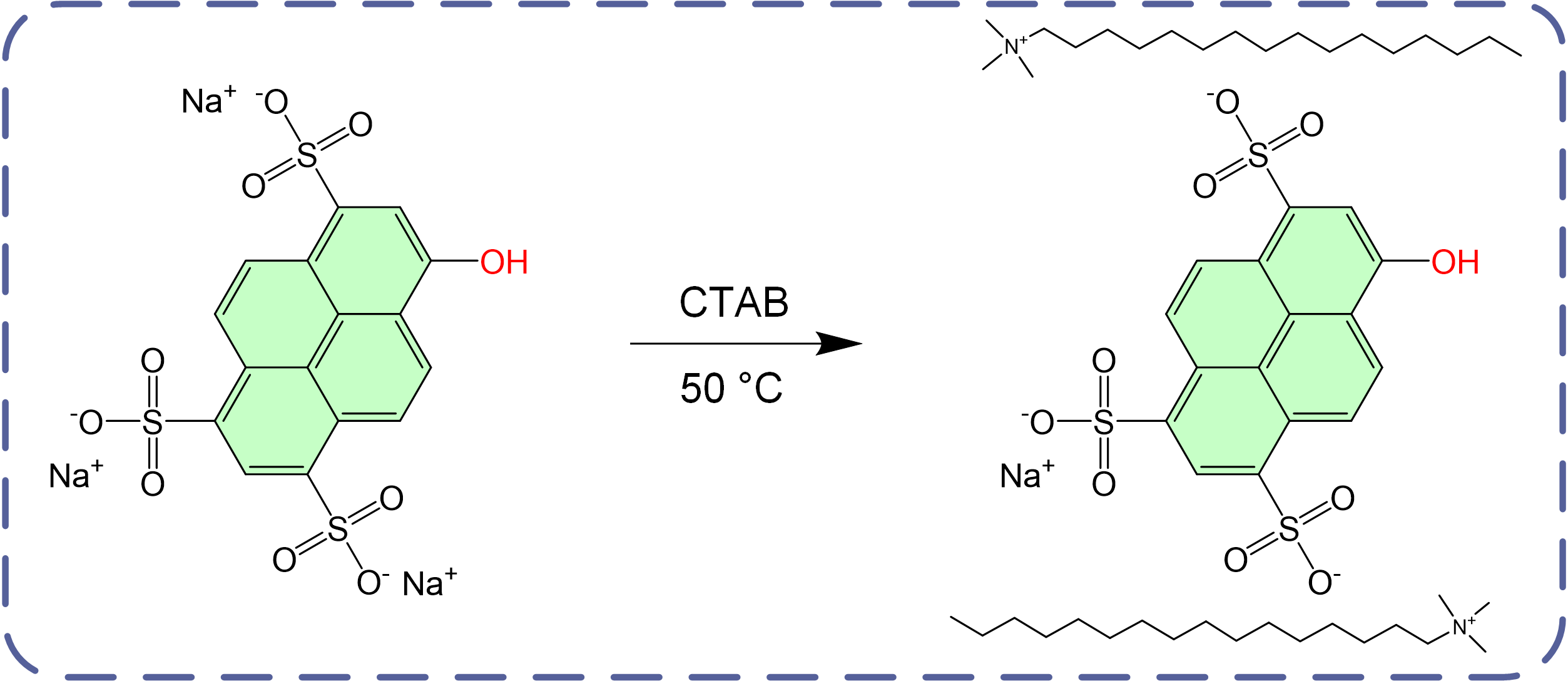


**Fig. S3.** Schematic of the synthesis of HPTS-IP. The preparation steps of HPTS-IP: (1) 3.8 mmol CTAB was dissolved in 125 mL deionized water and heated at 50 °C to be completely dissolved. (2) 1.9 mmol HPTS was dissolved in 125 mL deionized water, and the resulting solution was added to the aforementioned CTAB solution. (3) The mixture was heated at 50 °C for 1 hour, during which significant precipitation was observed. (4) The precipitate was collected by filtration and vacuum-dried overnight at 50 °C to obtain HPTS-IP.

**Response times of pH and temperature sensing**


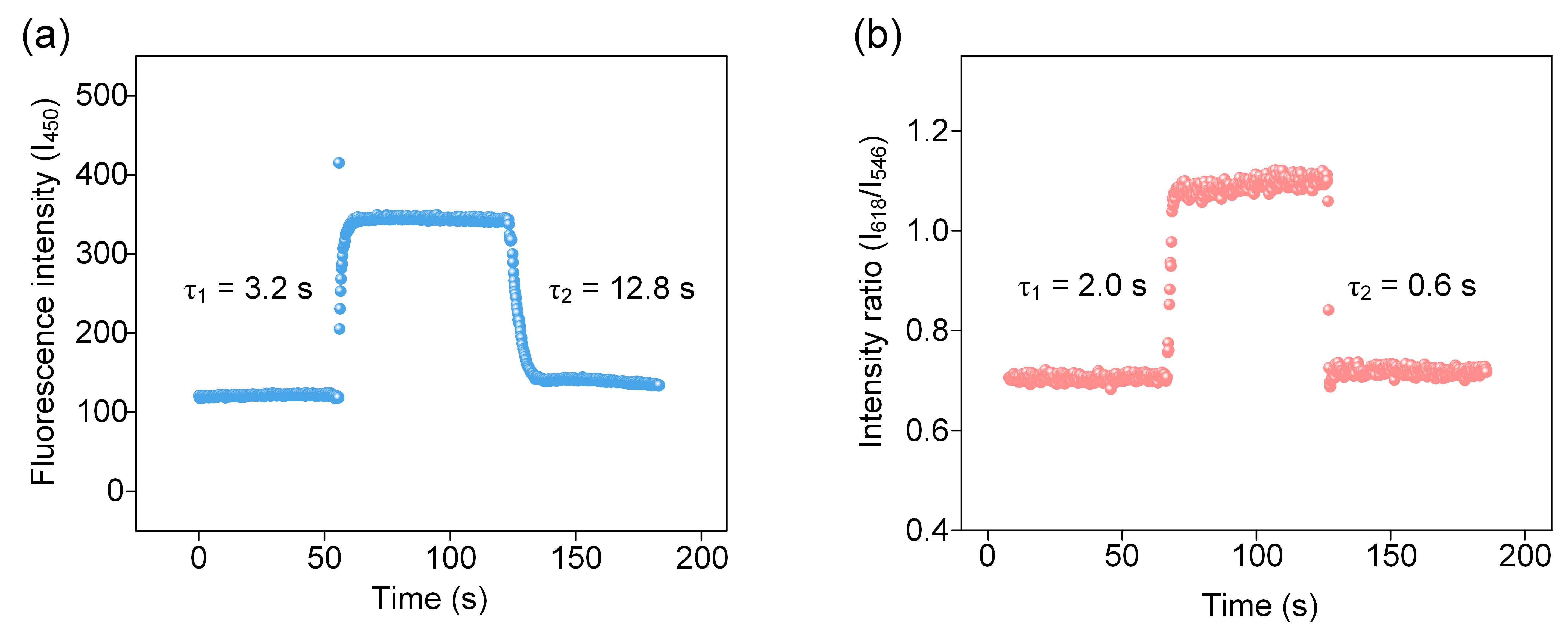


**Fig. S4.** Response times of (a) pH sensing with pH change from 6.0 to 8.0 and (b) temperature sensing with temperature change from 26 °C to 60 °C.

**Estimation of interference from autofluorescence**


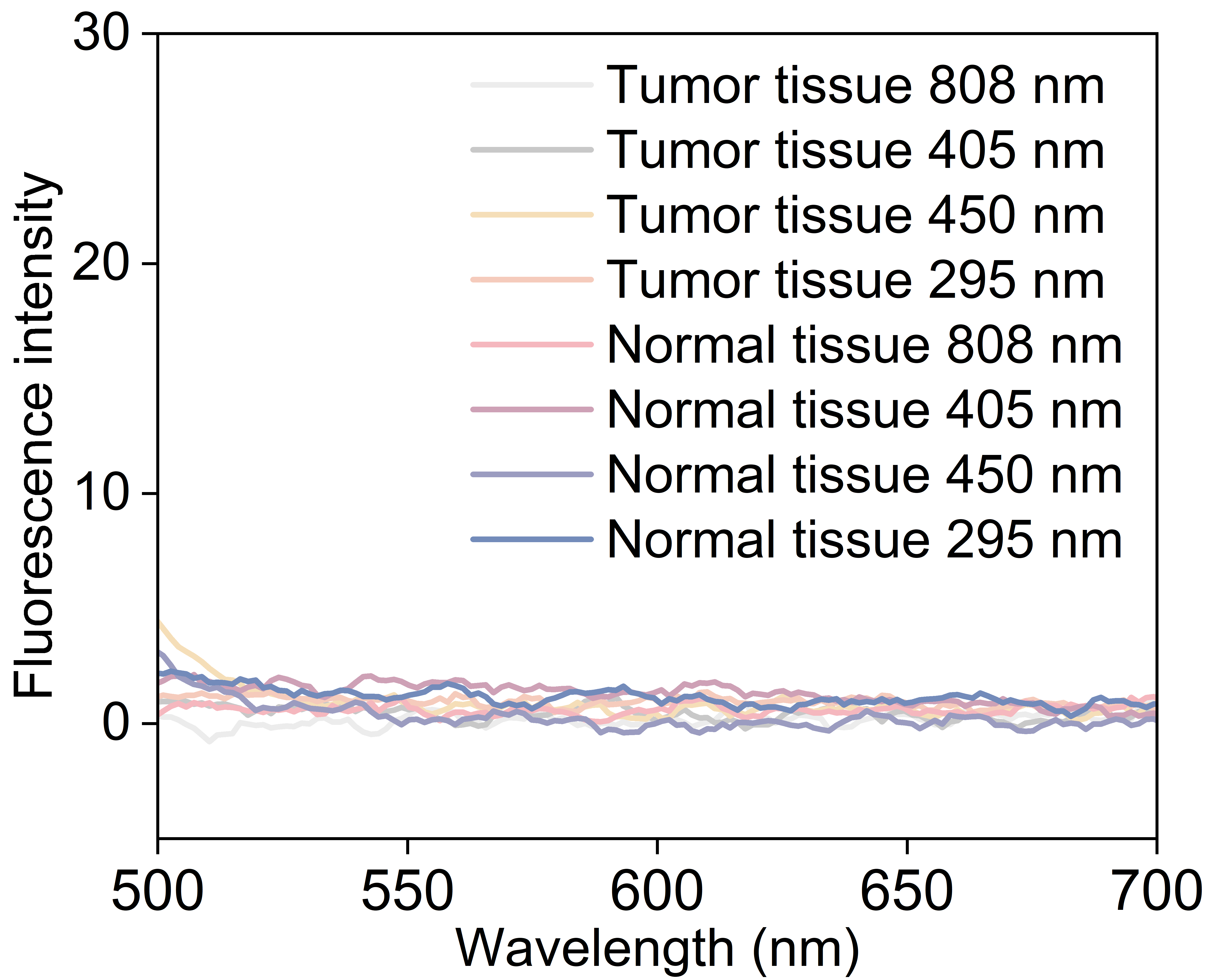


**Fig. S5.** Fluorescence spectra of the pure taper optical fiber (without the functional coating) in tumor tissue and normal tissue under 295 nm, 375 nm, 450 nm, and 808 nm excitation.

**Impact of 808 nm laser irradiation on sensing performance**


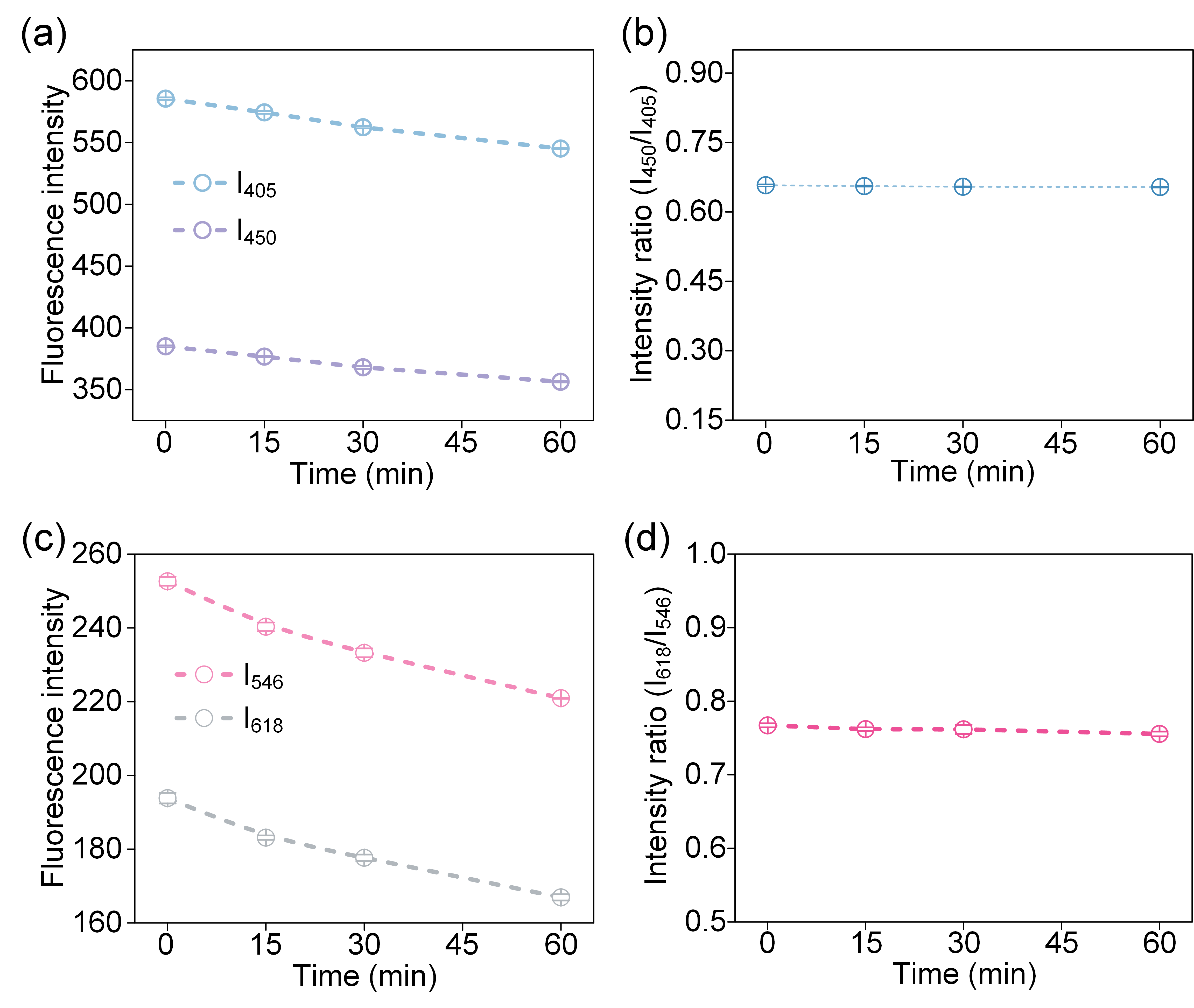


**Fig. S6.** Impact of 808 nm laser (500 mW) irradiation on sensing performance. (a-b) Changes in pH-sensing fluorescence intensity and intensity ratio as irradiation time increases. (c-d) Changes in temperature-sensing fluorescence intensity and intensity ratio as irradiation time increases.

**Absorption spectrums of ICG**

**
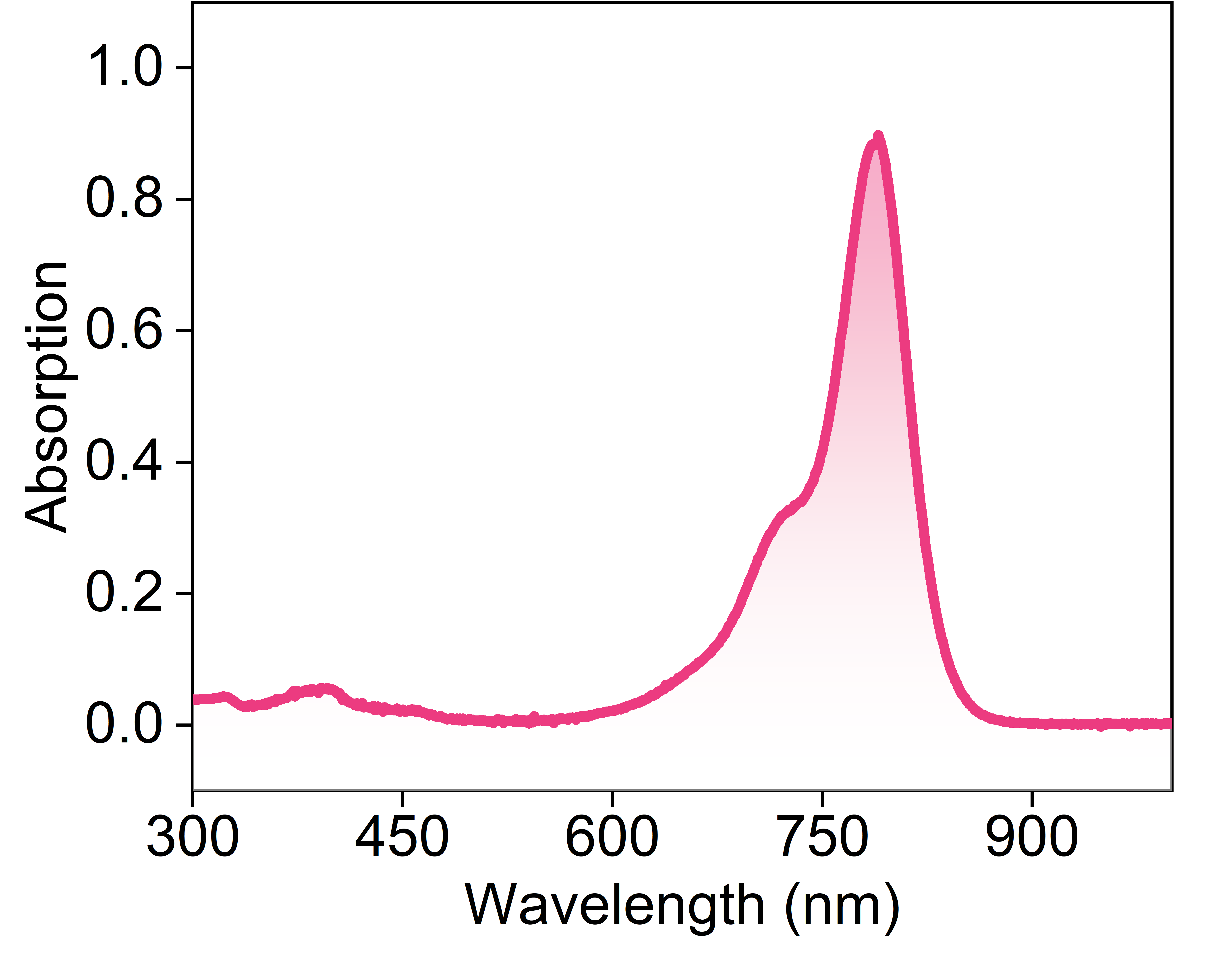
**

**Fig. S7.** Absorption spectrums of ICG in PBS solution (pH 7.4, 10 mM).

**MRI image analysis**

**
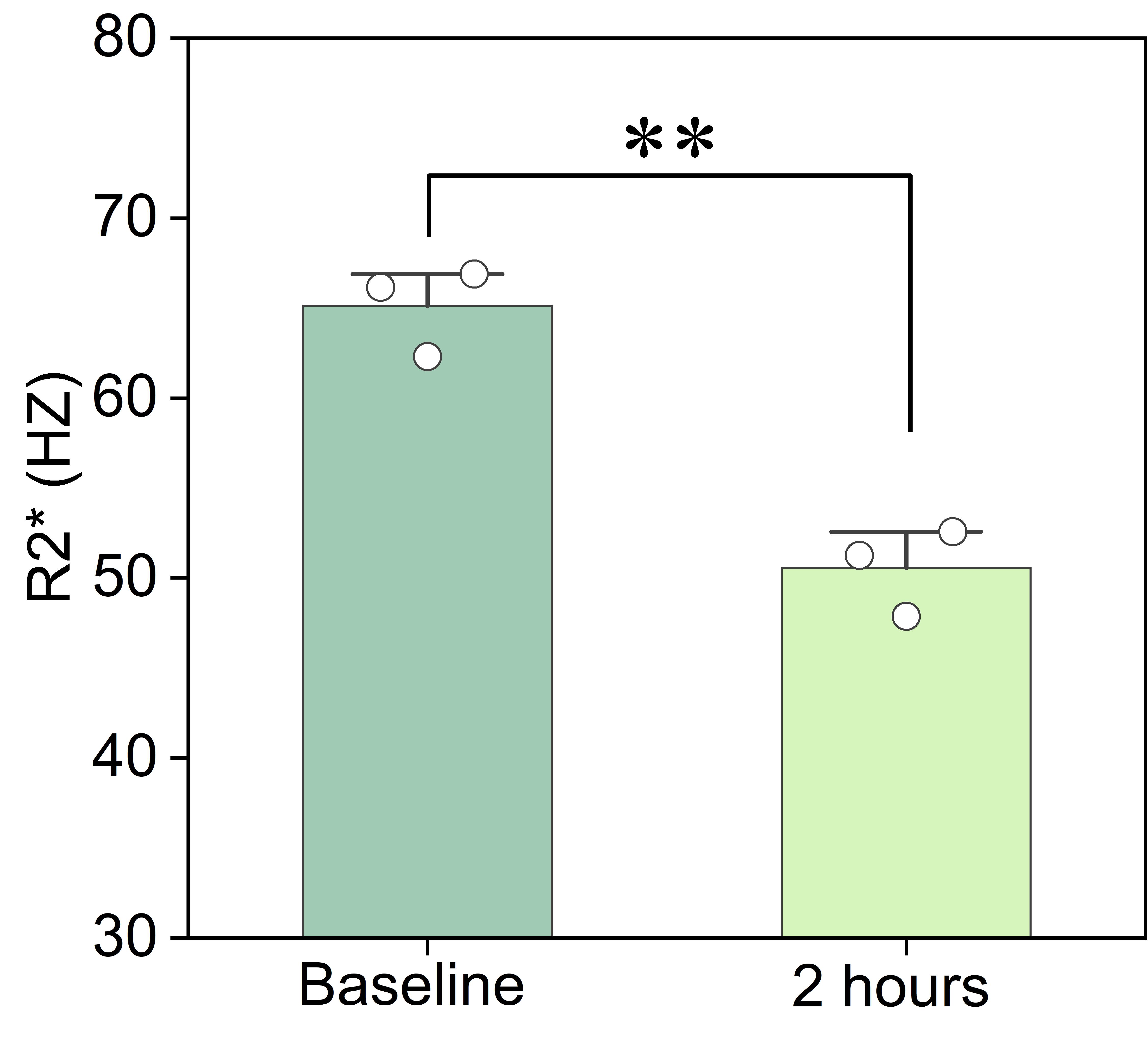
**

**Fig. S8.** MRI image analysis: comparison of tumors before (baseline) and 2 hours after treatment. A decrease in R2* value indicates a reduction in deoxyhemoglobin concentration and an elevation in tissue oxygenation level.

**CCK-8 assay**

**
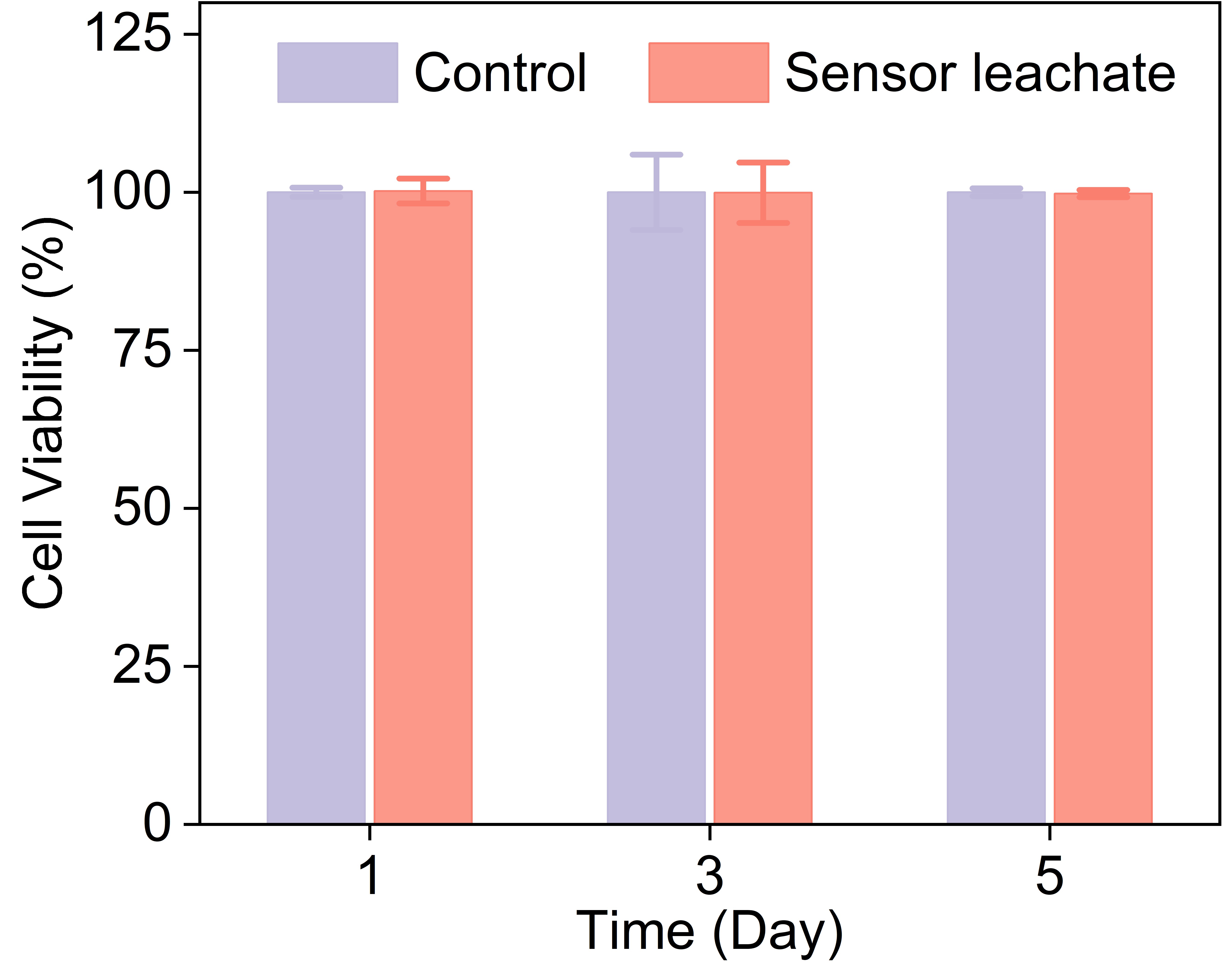
**

**Fig. S9.** The cell viability of HCT116 cells cultured with the cell culture medium soaked with the optical fiber probe for 24 hours (sensor leachate group) and with pure culture medium (control group) on days 1, 3, and 5.

**Organ biocompatibility**

**
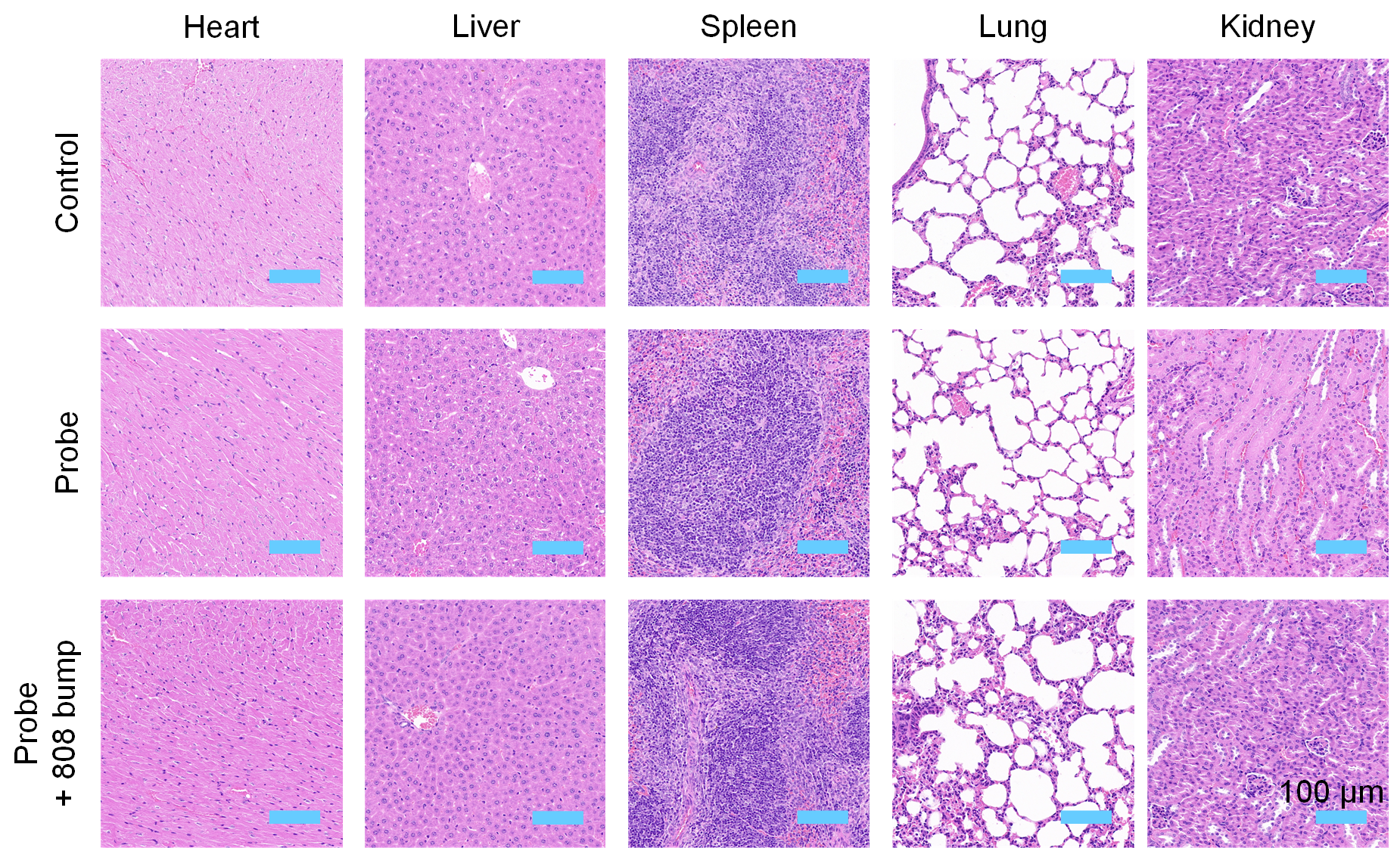
**

**Fig. S10.** H&E staining used to evaluate the effects of the fiber-optic probe on the organs. (In the probe+808 pump group, the fiber-optic probe was inserted into the tumor tissues of mice for 1 hour with excitation of 808 nm pump for 15 minutes, while the probe group without pump.

**Optimal concentrations of the three functional reagents**

**
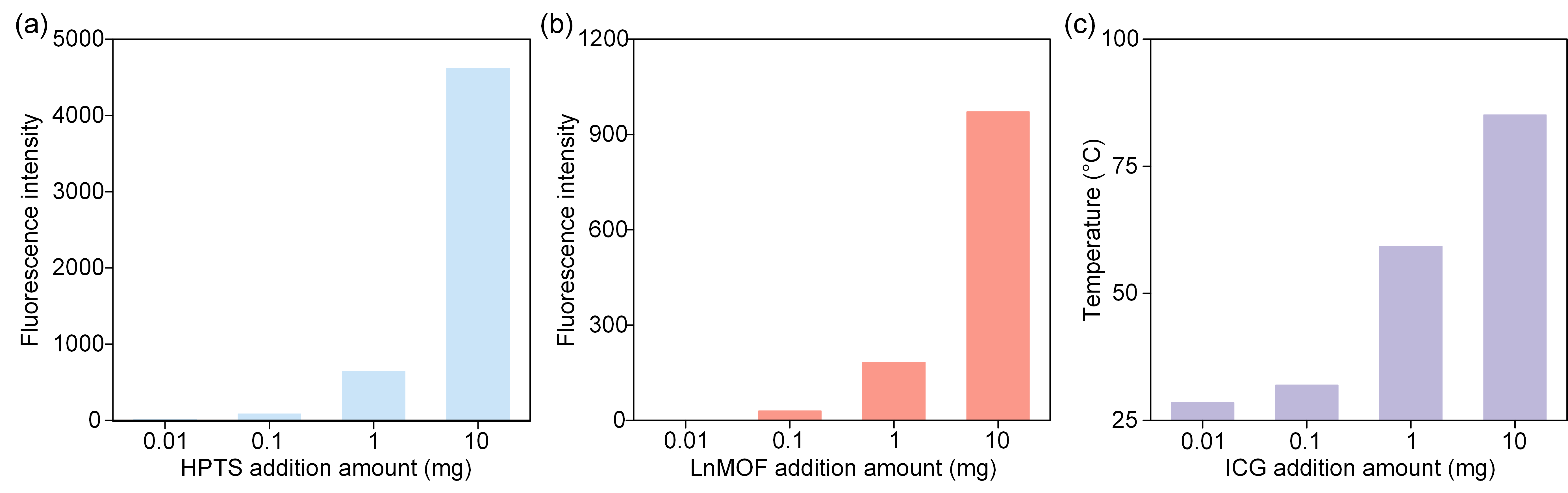
**

**Fig. S11.** Optimization experiments for the addition amount of the three functional reagents. (a) Comparison of fluorescence intensity at 520 nm under 450 nm excitation for fiber-optic probes with different HPTS addition amounts. (b) Comparison of fluorescence intensity at 546 nm under 295 nm excitation for fiber-optic probes with different LnMOF addition amounts. (c) Comparison of the maximum temperature at the fiber tip under 100 mW 808 nm laser excitation for fiber-optic probes with different ICG addition amounts. It should be underscored that excessively high concentrations of functional reagents could elevate the risk of biosafety issues due to potential reagents leaching. Therefore, for HPTS-IP and ICG, a loading amount of 1 mg was deemed sufficient to meet the requirements for in vivo theranostics, and higher concentrations were not adopted. In the case of LnMOF, a higher concentration (10 mg) was set, considering the significant transmission loss of the 295 nm excitation light in the optical fiber and the low output intensity of the wavelength-tunable light source.
